# Quantifying the Charge Carrier Interaction in Metallic Twisted Bilayer Graphene Superlattices

**DOI:** 10.3390/nano11051306

**Published:** 2021-05-15

**Authors:** Evgueni F. Talantsev

**Affiliations:** 1 M. N. Mikheev Institute of Metal Physics, Ural Branch, Russian Academy of Sciences, 18, S. Kovalevskoy St., 620108 Ekaterinburg, Russia; evgeny.talantsev@imp.uran.ru; Tel.: +7-912-676-0374; 2 Nanotech Centre, Ural Federal University, 19 Mira St., 620002 Ekaterinburg, Russia

**Keywords:** magic-angle twisted bilayer graphene, Moiré graphene superlattices, charge carrier interaction in two-dimensional materials, ε-phase of iron

## Abstract

The mechanism of charge carrier interaction in twisted bilayer graphene (TBG) remains an unresolved problem, where some researchers proposed the dominance of the electron–phonon interaction, while the others showed evidence for electron–electron or electron–magnon interactions. Here we propose to resolve this problem by generalizing the Bloch–Grüneisen equation and using it for the analysis of the temperature dependent resistivity in TBG. It is a well-established theoretical result that the Bloch–Grüneisen equation power-law exponent, p, exhibits exact integer values for certain mechanisms. For instance, *p* = 5 implies the electron–phonon interaction, *p* = 3 is associated with the electron–magnon interaction and *p* = 2 applies to the electron–electron interaction. Here we interpret the linear temperature-dependent resistance, widely observed in TBG, as p→1, which implies the quasielastic charge interaction with acoustic phonons. Thus, we fitted TBG resistance curves to the Bloch–Grüneisen equation, where we propose that *p* is a free-fitting parameter. We found that TBGs have a smoothly varied *p*-value (ranging from 1.4 to 4.4) depending on the Moiré superlattice constant, *λ*, or the charge carrier concentration, *n*. This implies that different mechanisms of the charge carrier interaction in TBG superlattices smoothly transition from one mechanism to another depending on, at least, *λ* and *n*. The proposed generalized Bloch–Grüneisen equation is applicable to a wide range of disciplines, including superconductivity and geology.

## 1. Introduction

Specific twist alignment of two single layer graphene (SLG) sheets at so-called magic rotation angles, *θ*, results in a Moiré superlattice with a larger period, *λ*, than the original SLG lattice [1,2]:

(1) $$\lambda =\frac{a}{2\cdot sin\left(\frac{\theta }{2}\right)},$$

where *a* = 0.246 nm is SLG lattice constant, and the magic angle *θ* is given by [2]:

(2) $$\theta =arccos\left(\frac{k^{2}+4\cdot k\cdot l+l^{2}}{2\cdot \left(k^{2}+k\cdot l+l^{2}\right)}\right),$$

where *l* and *k* are integers.

These twisted bilayer graphene (TBG) superlattices represent versatile two-dimensional (2D) materials in which depend on the rotation angle, *θ*, and change carrier doping, *n*, a wide number of physical effects, including superconductivity, can emerge [3,4,5,6,7,8,9,10,11,12,13,14,15]. It should be noted that recently Park et al. [16] experimentally found that magic-angle twisted trilayer graphene (MATTBG) superlattices also have correlated electronic states similarly to the ones observed in their bilayer counterparts.

One of the most important problem in understanding of all TBGs is the mechanism of the charge carrier interaction, where some research groups proposed that there is a dominant role of the electron–phonon interaction (which is also considered as the emerging mechanism for the superconductivity in MATBG by several research groups [17,18,19,20,21,22]), while the other groups showed evidence for the prevalence of the electron–electron interaction [3,10,16,23], and recently, new experiments demonstrated the dominance of the electron–magnon interaction [6,9,24,25]. It should be noted that Kerelsky et al. [26] reported that the superconducting state in TBG emerges at the same doping levels, *n*, and the twist angles, *θ*, at which the electron–electron interaction reaches its maximal values.

There is also an open question about the superconducting gap symmetry in TBG superlattices. If early research reports proposed an exotic *d* + *id* superconducting gap symmetry in TBG [3,27], more recently a classical *s*-wave [17,18,22] and an exotic *p*-wave [22,28] gap symmetries were proposed as well. More details on the superconducting properties of TBG can be found elsewhere [29].

Such a variety of proposed interaction mechanisms and mechanisms for the emergence of the superconducting state in TBGs reflect a large variety of physical effects which simultaneously can synergize in these 2D materials.

In attempting to quantify the charge carrier interaction effects in metallic TBG superlattices here, we proposed to use a generalized form of the Bloch–Grüneisen (BG) equation [30,31]. This equation was applied to the analysis of temperature dependent resistance in metallic TBG superlattices.

## 2. Proposed Model

The Bloch–Grüneisen (BG) equation [30,31] describes temperature dependent resistance in metallic compounds, and in its classical form, it can be written as

(3) $$R\left(T\right)=R_{0}+A_{1}\cdot T+\sum_{p}^{2,3,5}A_{p}\cdot {\left(\frac{T}{T_{\theta }}\right)}^{p}\cdot \int_{0}^{\frac{T_{\theta }}{T}}\frac{x^{p}}{\left(e^{x}-1\right)\cdot \left(1-e^{-x}\right)}\cdot dx,$$

where $R_{0}$ is the resistance at T→0 K, $T_{\theta }$ is the Debye temperature, $A_{p}$ is weighting parameters, and *p* is the power-law exponent, which has exact theoretical integer values for certain interaction mechanisms [32,33]:

(4) $$p=\{\begin{array}{l} 2\;\;implies\;that\;R\left(T\right)\;emerges\;from\;electron-electron\;interaction\\ 3\;\;implies\;that\;R\left(T\right)\;emerges\;from\;electron-magnon\;interaction\\ 5\;\;implies\;that\;R\left(T\right)\;emerges\;from\;electron-phonon\;interaction \end{array}$$

However, it should be noted that entire BG integral (Equation (2)) has a linear limit for p→1:

(5) $$\underset{p\to 1}{\lim}{\left(\frac{T}{T_{\theta }}\right)}^{p}\cdot \overset{\frac{T_{\theta }}{T}}{\underset{0}{\int }}\frac{x^{p}}{\left(e^{x}-1\right)\cdot \left(1-e^{-x}\right)}\cdot dx\to \left(\frac{B}{T_{\theta }}\right)\cdot T$$

where *B* is a constant. Thus, the linear term in Equation (3) can be also represented in the integral form with the weighting factor $A_{1}$:

(6) $$R\left(T\right)=R_{0}+\sum_{p}^{\left(\underset{p\to 1}{\lim}\right),2,3,5}A_{p}\cdot {\left(\frac{T}{T_{\theta }}\right)}^{p}\cdot \int_{0}^{\frac{T_{\theta }}{T}}\frac{x^{p}}{\left(e^{x}-1\right)\cdot \left(1-e^{-x}\right)}\cdot dx,$$

It should be noted that Equation (3) in its full form has been never applied to the analysis of experimental *R*(*T*) data, because the sum of strongly non-linear integrals has over-parametrization problem. Moreover, the majority of all published works utilizes Equation (3) where only electron–phonon integrand, i.e., *p* = 5, is included [34,35,36].

One of the possible ways to use an analytic power of Equation (3) is to reduce the number of integrals to one, but the use of the power-law exponent *p* will be the free-fitting parameter

(7) $$R\left(T\right)=R_{0}+A_{p}\cdot {\left(\frac{T}{T_{\theta }}\right)}^{p}\cdot \int_{0}^{\frac{T_{\theta }}{T}}\frac{x^{p}}{\left(e^{x}-1\right)\cdot \left(1-e^{-x}\right)}\cdot dx,$$

If the fit of *R*(*T*) to Equation (7) converges, then the deduced free-fitting parameter *p* should indicate the main charge carrier scattering mechanism in given materials.

To the author’s best knowledge, the approach to implement Equation (7) has been reported only by Jiang et al. [33] for Sr_2_Cr_3_As_2_O_2_, where the dominant role of the electron–magnon scattering (i.e., *p* = 3.34 [33]), with an insignificant part of the electron–phonon interaction (*p* = 5), has been revealed.

It should be noted that a replacement of the full integrals in Equation (3) or the integral in Equation (7) by power law terms

(8) $$R\left(T\right)=R_{0}+A_{p}\cdot T^{p},$$

which has been implemented in several reports [37,38,39], cannot be accepted to be accurate approximation, as it will be shown below herein.

As we mentioned above, another important feature of the Equation (6) is that the linear dependence of *R*(*T*) (or p→1 in terms of Equation (7)) in TBG has been proposed to be related to quasielastic scattering on acoustic phonon in MATBG [19], and thus, deduced *p*-values in the range of 1 < *p* < 2 have a clear interpretation as a sum of the electron–electron and electron–quasielastic acoustic phonon interactions.

**Figure 1:**
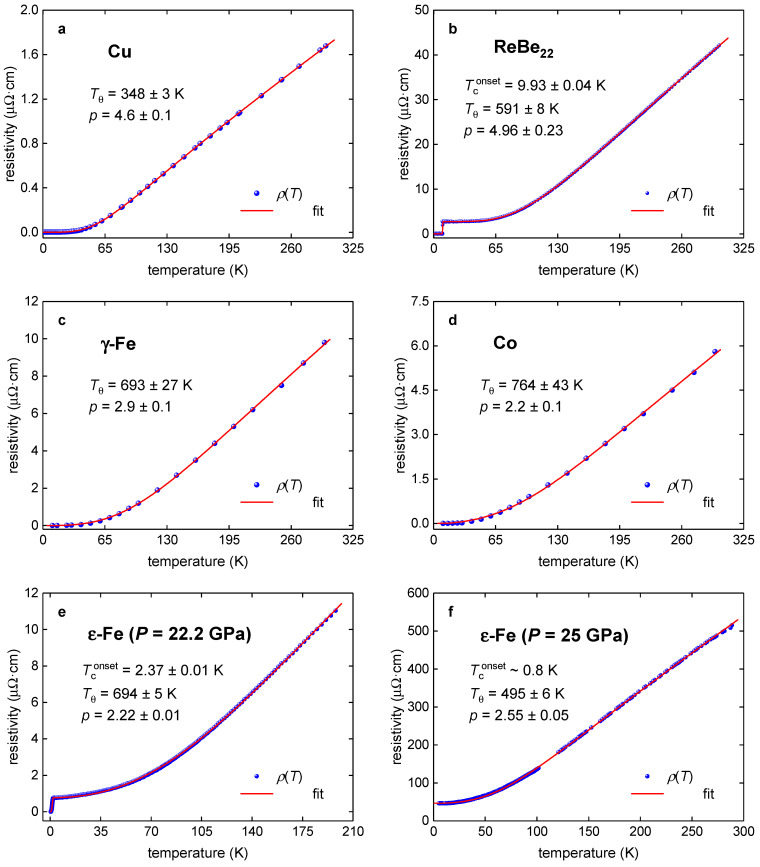
*ρ*(*T*) data and fits to generalized Bloch–Grüneisen (BG) equation (Equations (7) and (9)) for (**a**) pure Cu; (**b**) ReBe_22_; (**c**) pure ferromagnetic γ-Fe; (**d**) pure ferromagnetic Co; and (**e**,**f**) pure non-ferromagnetic highly-compressed ε-Fe. The raw data are reported in [34,37,40,42,44]. The red is the fitting curve, and 95% confidence bands are shown by a pink shaded area. Goodness of fit for all plots is better than *R* = 0.9990.

Here, we implemented Equation (7) to fit the *R*(*T*) data in TBG superlattices. First of all, we tested the validity of Equation (7) to be a proper fitting tool for classical electron–phonon materials, including electron–phonon mediated superconductors, from which we chose ReBe_22_ [34], as well as normal metal copper, and ferromagnetic iron and cobalt (for pure metals raw *R*(*T*) data were taken from the classical papers by White and Woods [40] and by Matula [41]), as well as for highly-compressed ε-phase of iron, which exhibits the superconducting state (for which raw *R*(*T*) data were reported by Shimizu et al. [42] and by Jaccard et al. [37]. In Figure 1, we show *R*(*T*) data and data fits for these materials. It should be noted that fits for superconducting ReBe_22_ and ε-Fe iron were performed by the recently proposed equation [43],

(9) $$R\left(T\right)=R_{0}+\theta \left(T_{c}^{onset}-T\right)\left(\frac{R_{norm}}{{\left(I_{0}\left(F\cdot {\left(1-\frac{T}{T_{c}^{onset}}\right)}^{3/2}\right)\right)}^{2}}\right)+\theta \left(T-T_{c}^{onset}\right)(R_{norm}+\;A\cdot \left({\left(\frac{T}{T_{\theta }}\right)}^{p}\cdot \int_{0}^{\frac{T_{\theta }}{T}}\frac{x^{p}}{\left(e^{x}-1\right)\left(1-e^{-x}\right)}dx-{\left(\frac{T_{c}^{onset}}{T_{\theta }}\right)}^{p}\cdot \int_{0}^{\frac{T_{\theta }}{T_{c}^{onset}}}\frac{x^{p}}{\left(e^{x}-1\right)\left(1-e^{-x}\right)}dx\right))$$

but where now we changed the *p*-value to be a free-fitting parameter, and where $T_{c}^{onset}$ is a free-fitting parameter of the onset of superconducting transition, *R*_norm_, is the sample resistance at the onset of the transition, *θ*(*x*) is the Heaviside step function, $I_{0}\left(x\right)$ is the zero-order modified Bessel function of the first kind, and *F* is a free-fitting dimensionless parameter.

*R*(*T*) data fits to Equations (7) and (9) have been performed by utilizing the Levenberg–Marquardt algorithm in non-linear fitting package of the Origin software (ver. 2017, Origin Lab, Northampton, MA, USA).

## 3. Results

### 3.1. Pure Metals and Binary Alloy ReBe_22_

First, to prove the validity of the approach, we applied Equations (7) and (9) for pure metals and the binary alloy ReBe_22_ (Figure 1) to compare the deduced value with a theoretically calculated one. The theory [30,31] predicts that *R*(*T*) dataset of pure perfect nonferromagnetic metals should be described by Equation (7) with *p* = 5. In Figure 1a we showed the fitted *R*(*T*) dataset (reported by Teixeira [41,44] for pure copper) where deduced *p* = 4.6 ± 0.1 and *T_θ_* = 348 ± 3 K. *p*-value is reasonably close to the theoretical value of *p* = 5, that was calculated [30,31,40] in an assumption of a free-electron model in defect-free metal. In Figure 1b one can see that expected *p* = 5 (which implies the dominance of the electron–phonon interaction) has been revealed for the electron–phonon mediated ReBe_22_ superconductor.

Our analysis revealed that γ-Fe (Figure 1c), which should have *p* = 3 [32,33,40,41], exhibits *p* = 2.9 ± 0.1, which is an excellent demonstration of the applicability of Equation (7) to the analysis. Ferromagnetic cobalt has *p* = 2.2 ± 0.1, which reflects a well-established fact that the electron–electron interaction in this element plays a significant role [40].

Another interesting result, which shows the validity of the approach for a much wider class of the materials, was obtained for hexagonal-close-packed highly-compressed iron, ε-Fe. It should be stressed that this ε-Fe phase plays a crucial role in the Earth geology [37,39] and thus, our approach can potentially impact a broad range of disciplines beyond 2D materials. Truly, the electrical conductivity, ρρ, is directly linked with the heat transfer due to the Wiedemann–Franz law:

(10) $$k\left(T,P\right)=\frac{L\cdot T}{\rho \left(T,P\right)},$$

where *k* is the thermal conductivity, and *L* = 2.44⋅10^−8^ WΩK^−2^ is the Lorenz number, and *P* is the pressure. Due to all Earth’s planetary models which are based on the assumption that the Earth crust is formed by ε-Fe, the validity of that model crucially depends on the accuracy of the utilized ρ(T,P) function, for which the integral form of Equation (7) (vs the power-law utilized in Equation (8)) provides the best accuracy (details can be found in [39]).

To the best of the author’s knowledge, to date, the experimental ρ(*T*,*P*) data for ε-Fe were fitted only to an approximant function of Equation (7), i.e., Equation (8). In a result, the reported *p* values are within an extremely wide range of *p* = 1.5–5.9, and moreover, we found herein that the approach to use Equation (8) leads to wrong *p*-values. Truly, in Figure 1e, we show the fit to Equation (7), which reveals *p* = 2.22 ± 0.01 for which, by employing the same *ρ*(*T*) dataset and the use of Equation (10), Jaccard et al. [37] reported *p* = 1.67. If our value of *p* = 2.22 ± 0.01 shows that electric charge carriers in ε-Fe phase exhibit two scattering mechanisms (i.e., mainly the electron–electron interaction (*p* = 2) with some weighting part of the electron–magnon interaction (*p* = 3)), the interpretation for *p* = 1.67 reported by Jaccard et al. [37] cannot be founded, because p→1 case is only applicable for MATBG superlattices [20], and *p*-values below 2 are simply prohibited for elemental metals, because there is no physical interpretation for such values.

It is important to note that there is a nice correlation between *p*-values and superconducting transition temperatures, *T*_c_, in ε-Fe phase too. If for *p* = 2.55 ± 0.05 (which implied a significant electron–magnon interaction) the full resistive transition does not occur (and where the only 10% drop in resistance is observed, with the onset of transition temperature, $T_{c}^{onset}\sim 1\;K$), for ε-Fe sample, for which *p* = 2.22 ± 0.01 was revealed, the full resistive transition was observed with $T_{c}^{onset}=2.37\pm 0.01\;K$. This result has a clear interpretation that the suppression of the electron–magnon interaction causes the formation of more robust superconducting condensate.

### 3.2. SLG/hBN Superlattice

**Figure 2:**
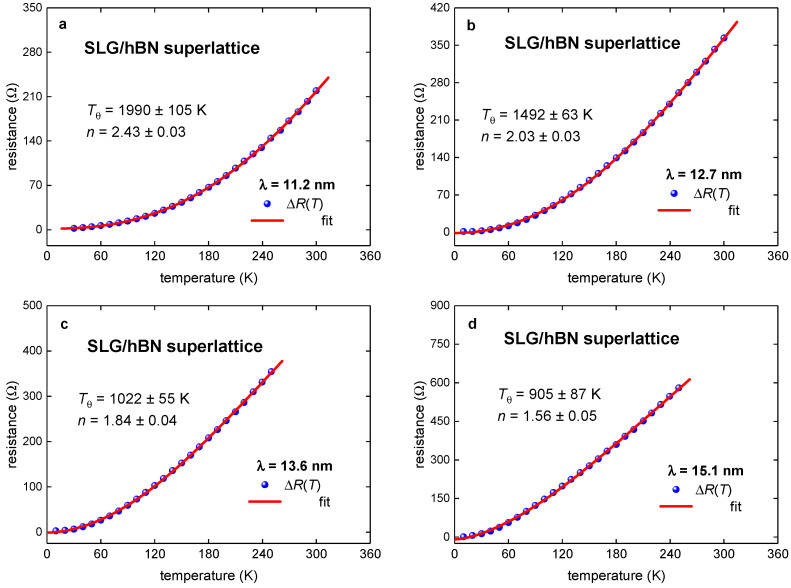
*R*(*T*) data for Moiré SLG/hBN superlattices (raw data reported by Wallbank et al. [10]) and fits to Equation (8) for (**a**) *λ* = 11.2 nm, (**b**) *λ* = 12.7 nm, (**c**) *λ* = 13.6 nm, and (**d**) *λ* = 15.1 nm. Red lines are the fitting curves; 95% confidence bands are shown by a pink shaded area. Goodness of fit for all plots is better than *R* = 0.9997.

**Figure 3:**
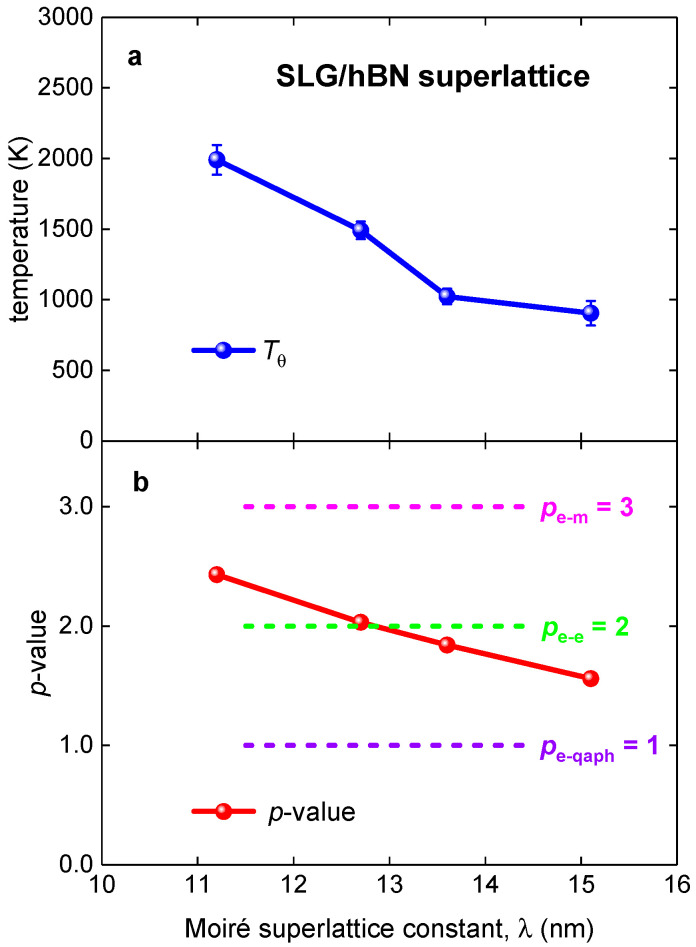
Summarized results for Moiré SLG/hBN superlattices (raw data reported by Wallbank et al. [10]). (**a**) deduced Debye temperature; (**b**) deduced *p*-value in Equation (7). Characteristic values for the quasielastic electron–acoustic phonon interaction (*p*_e-qaph_ = 1), the electron–electron interaction (*p*_e-e_ = 2), and the electron–magnon interaction (*p*_e-m_ = 3) are shown.

Now, we turn to the analysis of TBG superlattices. First, we analyzed the experimental R(T) curves for the Moiré superlattice in single layer graphene on the hBN single crystal (SLG/hBN superlattice) reported by Wallbank et al. [10], where the Moiré superlattice constant, λ, has been changed in the range of *λ* = 11.2—15.1. Fits to Equation (7) are shown in Figure 2 and summarized results in Figure 3. Overall, our analysis confirms the result reported by Wallbank et al. [10] that the electron–electron interaction is dominant in these Moiré 2D superlattices. However, our analysis shows a smooth and near-linear dependence of *p*-value and the Debye temperature, T_θ_, on the Moiré superlattice constant, *λ* (Figure 3).

If for conventional conductors the linear *R*(*T*) dependence is interpreted as an approximation of the Equation (7) with *p* = 5 and $T>T_{\theta }$ [40,41], for TBG superlattices the linear *R*(*T*) dependence in term Equation (7) has different interpretation as a case of pure electron–quasielastic acoustic phonon (e-qaph) interaction [19].

Thus, if TBG superlattice exhibits 1 < *p* < 2 (see, for instance, Figure 2 and Figure 3), these *p*-values can be interpretated as a manifestation of intermediate TBG state between the pure electron–electron charge carrier interaction (e-e), for which the characteristic value is *p* = 2, and the pure electron–quasielastic acoustic phonon interaction (e-qaph), for which the characteristic value is *p* = 1.

### 3.3. WSe_2_/TBG/WSe_2_ Superlattice

**Figure 4:**
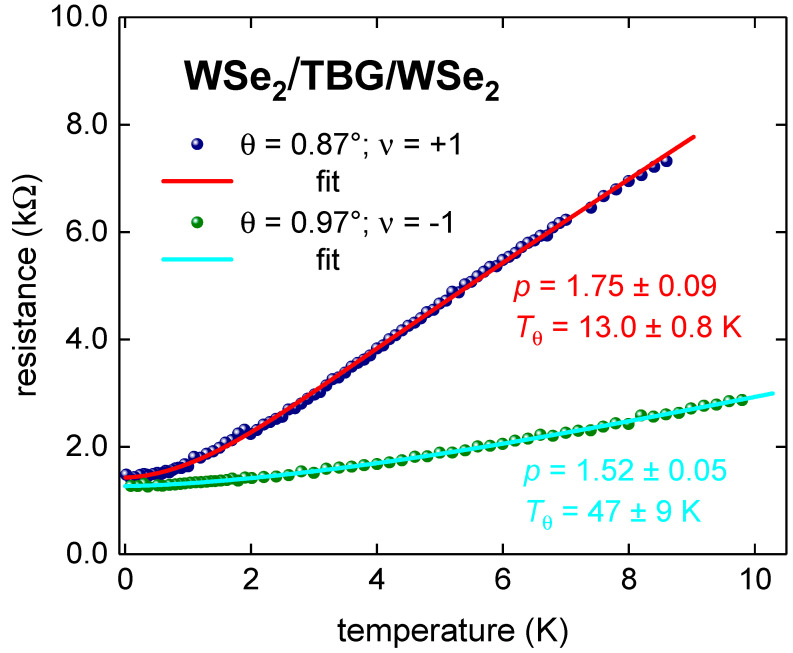
*R*(*T*) fits to Equation (7) for WSe_2_/TBG/WSe_2_ (raw data reported by Arora et al. [11]) for *θ* = 0.87° and *θ* = 0.97°. 95% confidence bands are shown. Goodness of fit is better than *R* = 0.998.

The similar mixed state characterized by 1 < *p* < 2 has been also revealed in the metallic TBG superlattice stabilized by WSe_2_, i.e., WSe_2_/TBG/WSe_2_, for which raw *R*(*T*) data were reported by Arora et al. [11]. In Figure 4, we show *R*(*T*) data and fits for samples with twisted angles *θ* = 0.87° (filling factor *ν* = +1, deduced *p* = 1.52 ± 0.05, *T_θ_* = 47 ± 9 K) and 0.97° (filling $\nu =-1_{\theta }$, deduced *p* = 1.75 ± 0.09, *T_θ_* = 13.0 ± 0.8 K).

### 3.4. TBG Superlattice with θ = 2.02°

Most experimental studies in TBG superlattices have been performed for the *R*(*T*) dependences on the charge carrier density, *n*. In this work, we performed the analysis for the metallic states of TBG system which exhibits the twisted angle of *θ* = 2.02° (for which the raw experimental *R*(*T*) was reported by Polshyn et al. [8]). The data were analyzed in the full range of the charge carrier density of $n\leq \pm \left|6.71\right|\cdot 10^{12}\;{\mathrm{cm}}^{-2}$.

**Figure 5:**
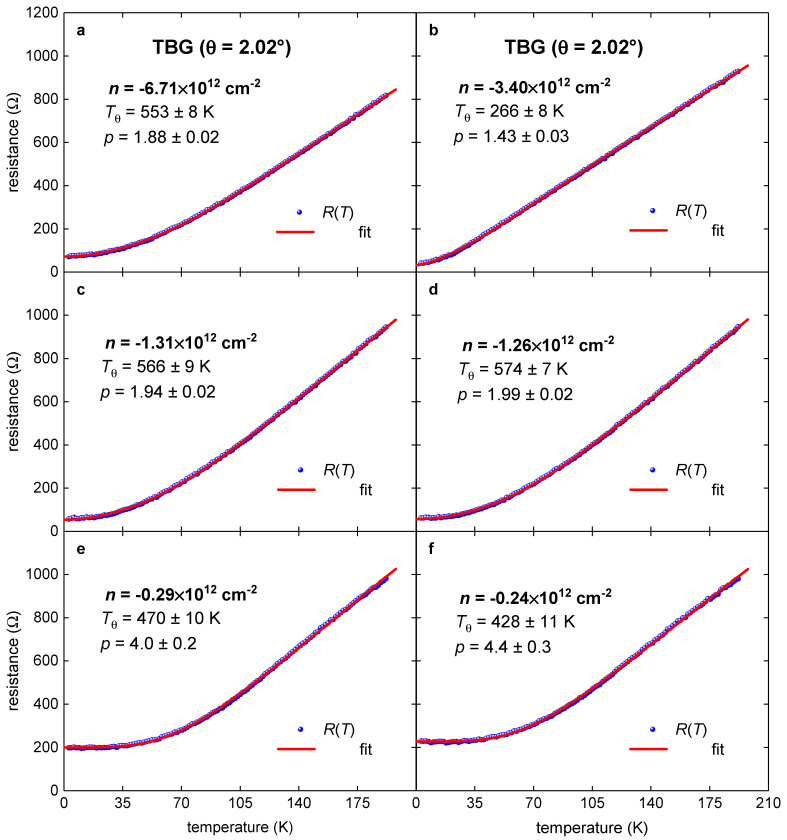
*R*(*T*) data and data fit to Equation (7) for metallic TBG superlattice on hole side with *θ* = 2.02° (raw *R*(*T*) data were reported by Polshyn et al. [8]). The doping state, *n*, for this TBG superlattice gradually varies from $n=-6.71\cdot 10^{12}\;{\mathrm{cm}}^{-2}$ (**a**) to $n=-0.24\cdot 10^{12}\;{\mathrm{cm}}^{-2}$ (**f**). Red are the fitting curves; 95% confidence bands are shown by a pink shaded area. Goodness of fit for both plots is better than *R* = 0.9990.

**Figure 6:**
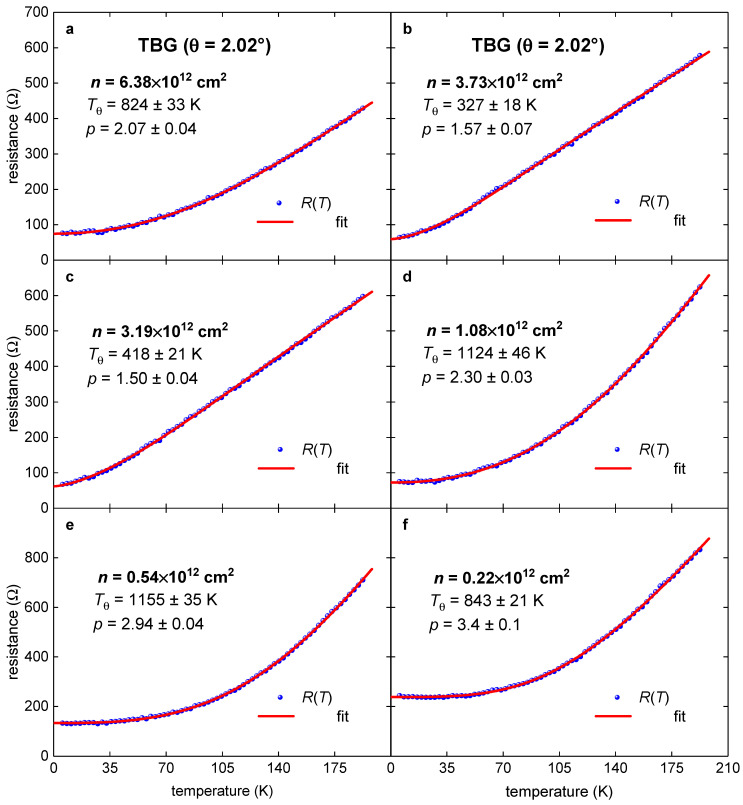
*R*(*T*) data and data fit to Equation (7) for metallic TBG superlattice on electron doping side with *θ* = 2.02° (raw *R*(*T*) data were reported by Polshyn et al. [8]). The doping state, *n*, for this TBG superlattice gradually varies from $n=6.38\cdot 10^{12}\;{\mathrm{cm}}^{-2}$ (**a**) to $n=0.22\cdot 10^{12}\;{\mathrm{cm}}^{-2}$ (**f**). Red are the fitting curves; 95% confidence bands are shown by a pink shaded area. Goodness of fit for both plots is better than *R* = 0.9990.

We presented herein the results for *R*(*T*) data analysis which was undertaken at *T* ≤ 192.5 K. Representative fittings where *p*-value is reaching the characteristic values of *p* = 2, *p* = 3, as well as a low value of *p* = 1.4 and the highest value of *p* = 4.7 are shown in Figure 5 and Figure 6. We do not fit *R*(*T*) curves measured at very low charge carrier density, because these curves have a low-temperature upturn in the *R*(*T*) which indicates the transition into a semiconductor or insulating state.

## 4. Discussion

**Figure 7:**
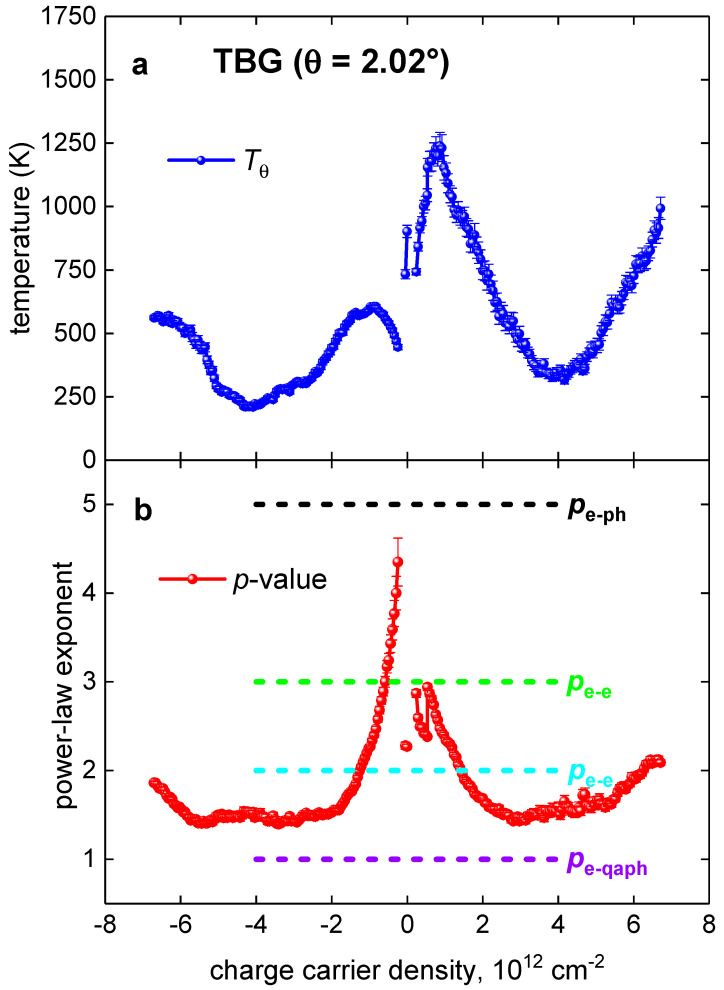
Summarized results for TBG superlattice with *θ* = 2.02°. (**a**) Deduced Debye temperature; (**b**) deduced *p*-value. Characteristic values for the quasielastic electron–acoustic phonon interaction (*p*_e-qaph_ = 1), the electron–electron interaction (*p*_e-e_ = 2), the electron–magnon interaction (*p*_e-m_ = 3), and the electron–phonon interaction (*p*_e-ph_ = 5) are shown.

The reported results for the Debye temperature, *T_θ_*(*p*), and the power-law exponent, *p*(*n*), vs. the charge carrier density for this TBG superlattice is shown in Figure 7. There are several important findings:

1. A classical electron–phonon interaction (with *p* > 3.5) can be observed at the lowest charge carrier concentration in a very narrow concentration range, $-0.39\cdot 10^{12}\;{\mathrm{cm}}^{-2}<n<-0.39\cdot 10^{12}\;{\mathrm{cm}}^{-2}$. In this doping range, we where we skipped from the analysis several *R*(*T*) curves measured at some very low *p*, which exhibits an upturn in *R*(*T*) at *T* < 20 K.
2. A classical electron–phonon interaction (with *p* > 3.5) can be observed at the lowest charge carrier concentration in a very narrow concentration range, $-0.39\cdot 10^{12}\;{\mathrm{cm}}^{-2}<n<-0.39\cdot 10^{12}\;{\mathrm{cm}}^{-2}$. In this doping range, we where we skipped from the analysis several *R*(*T*) curves measured at some very low *p*, which exhibits an upturn in *R*(*T*) at *T* < 20 K.
3. The dominant role of the electron–magnon interaction (2.5 < *n* < 3.5) has been revealed at low charge carrier concentration, $\left|0.4\right|\cdot 10^{12}\;{\mathrm{cm}}^{-2}<n<\left|1.0\right|\cdot 10^{12}\;{\mathrm{cm}}^{-2}$. Physical interpretation of this result can be based on the recent reports [6,9,12,23,24,25] where it was shown that the ferromagnetic type of ordering does exist at some intermediate doping levels between the insulating and highly conductive TBG states.
4. In a wide range of doping, $\left|1.0\right|\cdot 10^{12}≲n≲\left|5.5\right|\cdot 10^{12}\;{\mathrm{cm}}^{-2}$, the interaction belongs to a sum of the electron–electron and the electron–quasielastic acoustic phonon interactions. This result can be understood if one considers that in a perfect crystalline 2D sheet the charge carriers exhibit two main interactions, the Coulomb retraction, and an interaction with the lattice vibrations. Which interaction becomes dominant depends on the details; however, there is a general trend that at some low *n,* the Coulomb retraction is also low because the charge carriers are well spatially separated from each other. Thus, relative strength of the charge carrier interaction with the lattice vibrations cannot be low if even this interaction is weak in its absolute value. However, as far as the doping level *n* is increasing, the Coulomb retraction is also increasing, and at some *n*-value, it becomes dominant. This is exactly what we reveal at the highest doping level, *n*, considered in this report.
5. At the highest charge carrier density, $n>\left|5.5\right|\cdot 10^{12}\;{\mathrm{cm}}^{-2}$, considered in this report, the electron–electron interaction overcomes the other interactions, and *p*-value towards 2, while the doping is increasing. This is due to the fact that the charge carrier concentration, *n*, becomes high, and the spatial charge separation reduces to the level when the Coulomb charge retraction becomes overwhelmingly strong in the comparison with other interactions.

## 5. Conclusions

In this paper, we aim to propose an approach to quantify the charge carrier integration in metallic materials by generalizing the Bloch–Grüneisen equation, where the power-law exponent is a free-fitting parameter. In the case of twisted bilayer graphene superlattices, we show that the interaction mechanism can be smoothly transformed from one to another by a variation of either the Moiré superlattice constant, λ, or the charge carrier concentration. We also show that generalized Bloch–Grüneisen equation can be an instructive tool to study different topics in natural science, including the Earth geology.

It is important to note that recently, a new fundamental property of the single layer graphene that can potentially be in use in the cosmology [45,46] has been discussed in the literature. This is a ground to expect that new unusual properties of twisted multilayered graphene Moiré superlattices will be further discovered.

The proposed approach potentially can be applicable for a wide range of materials, including 2D superconductors [47,48,49,50,51,52,53,54,55,56] and superhydrides [57,58,59,60,61,62,63,64,65].

## Data Availability

No new data were created or analyzed in this study. Data sharing is not applicable to this article.
